# Benign Adenomyoepithelioma of the Breast: A Case Report and Review of Imaging Features

**DOI:** 10.7759/cureus.58421

**Published:** 2024-04-16

**Authors:** Timea A Kovacs, Sophia N Mourad, Andrew Dakkak, Matthew Burger, Michele Edison

**Affiliations:** 1 Medicine, Florida State University College of Medicine, Pensacola, USA; 2 Medicine, Florida State University College of Medicine-Orlando Regional Campus, Orlando, USA; 3 Diagnostic Radiology, AdventHealth Orlando, Orlando, USA

**Keywords:** ame, breast mass, malignant adenomyoepithelioma, benign adenomyoepithelioma, adenomyoepithelioma

## Abstract

Adenomyoepithelioma (AME) of the breast is a rare tumor that can be benign or malignant and has varied morphological features. We report a case of a 62-year-old female with a history of right breast cancer who presented with abnormal screening mammography. The detection, presentation, and varied imaging characteristics of AMEs are discussed. The nonspecific imaging and histologic appearance of AME are highlighted, emphasizing the need for representative biopsy samples and histopathological review for diagnosis. Our case underlines the importance of wide surgical excision with negative margins in the presence of diagnostic uncertainty, which corresponds with the current recommended treatment for AME to prevent recurrence.

## Introduction

Adenomyoepithelioma (AME) is a rare primary breast tumor characterized by biphasic proliferation of epithelial and myoepithelial cells. AMEs have a variable clinical presentation and nonspecific imaging appearance. Although certain mammographic and ultrasonographic imaging features have been reported, a final diagnosis of AME requires biopsy and histopathological analysis to guide proper management. Herein, we present a case of AME and discuss the clinical presentation, imaging features, and pathology.

## Case presentation

**Figure 1:**
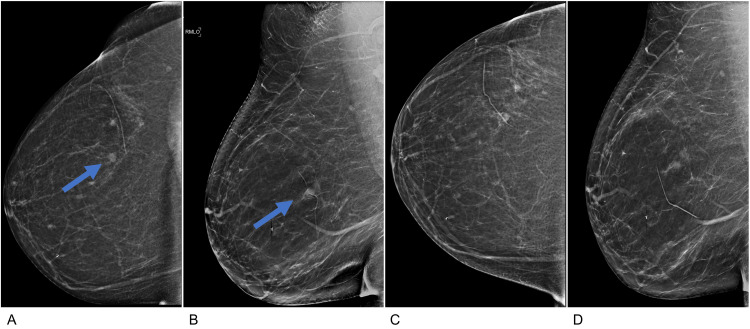
Screening mammogram Current screening mammogram synthesized 2D craniocaudal (A) and mediolateral oblique (B) images demonstrate scattered areas of fibroglandular density and a 0.8 cm developing focal asymmetry (blue arrow) in the right breast upper outer quadrant. Screening mammograms from one year prior, synthesized 2D craniocaudal (C), and mediolateral oblique (D) images are included for comparison. Note that a linear scar marker is present on the patient’s skin, indicating the site of a prior lumpectomy for breast cancer.

A 62-year-old female with a history of right breast cancer presents with a new 0.8 cm focal asymmetry in the upper outer quadrant right breast, detected on screening mammography (Figure 1A, 1B) in comparison to the prior year (Figure 1C, 1D).

**Figure 2:**
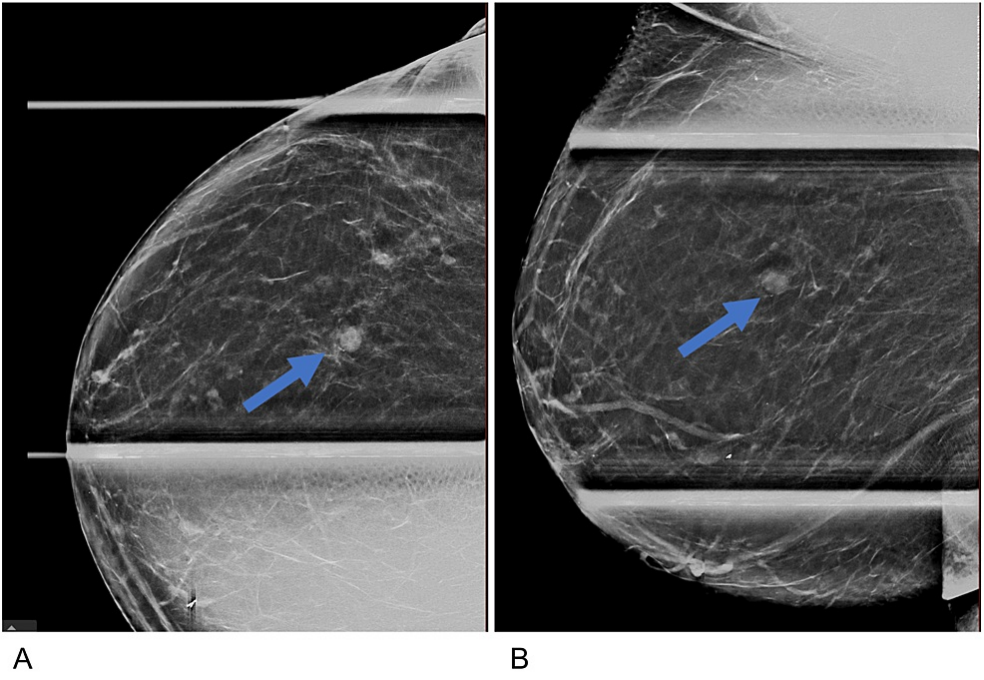
Right breast diagnostic mammogram Right breast diagnostic mammogram spot compression craniocaudal (A) and mediolateral oblique (B) images demonstrate a circumscribed round indistinct mass (blue arrow) in the upper outer quadrant right breast measuring 0.9 x 0.6 x 0.7 cm.

The mass was not palpable on clinical examination and had no associated signs or symptoms. The patient was recommended to return for a right diagnostic mammogram and targeted ultrasound. Spot compression digital tomosynthesis views of the upper outer quadrant right breast revealed an indistinct, round mass (Figure 2A, 2B). 

**Figure 3:**
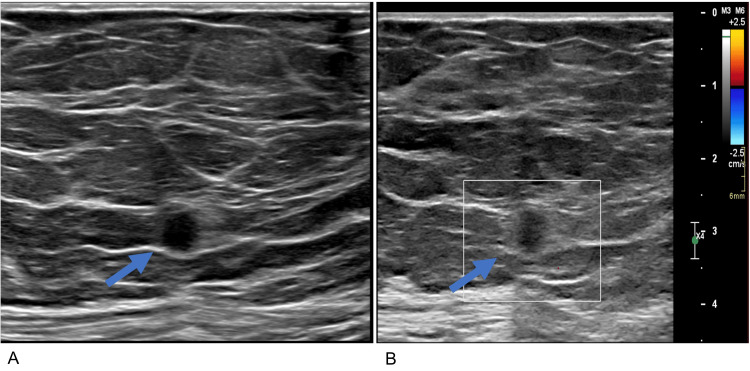
Ultrasound of the right breast Ultrasound imaging of the upper outer quadrant right breast grayscale (A) and color Doppler (B) images of the 9:00 axis 5 cm from the nipple show a round, indistinct, hypoechoic mass (blue arrow) without internal Doppler signal measuring 0.8 x 0.7 x 0.5 cm, which corresponds to the mammographic mass.

Ultrasound revealed a corresponding round, indistinct, hypoechoic mass with no internal vascularity measuring 0.8 x 0.7 x 0.5 cm at the right breast 9:00 axis 5 cm from the nipple (Figure 3A, 3B). Due to the suspicious imaging appearance, a right breast ultrasound-guided core needle biopsy was performed. Pathologic evaluation of the needle core biopsy resulted in atypical sclerosing ductal epithelial lesion with apocrine metaplasia.

**Figure 4:**
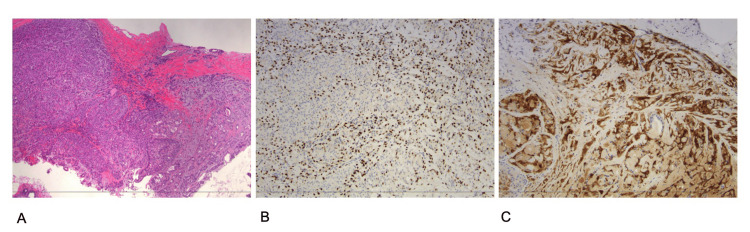
Pathology (A) Hematoxylin and eosin (H&E)-stained section of the adenomyoepithelioma (core biopsy) at 40x magnification. (B) S100 and p63 stains, used as myoepithelial cell markers in the breast, at 100x magnification. (C) Immunohistochemistry at 100x magnification demonstrating the many myoepithelial cells (dark brown staining). In normal breasts, we would expect only a single layer of myoepithelium around the inner ductal cells.

Histologic sections demonstrated a proliferative lesion characterized by probable apocrine differentiation, focally sclerotic stroma, and the presence of myoepithelial cells throughout portions of the lesion (Figure 4A). P63, S100, and CK5/6 were positive in myoepithelial cells and the majority of ductal epithelial cells (Figure 4B). Additional immunohistochemistry was positive for GATA3 and GCDFP15 in rare cells (Figure 4C). The differential diagnosis included sclerosing apocrine adenosis and AME with apocrine metaplasia. Complete surgical excision was recommended for definitive histopathologic subclassification. A SAVI Scout-localized right lumpectomy was performed on the 9:00 lesion for complete histologic characterization. The specimen was a 5.5 x 6.0 x 0.5 cm fragment of fibroadipose tissue. The specimen was sectioned from superior to inferior to reveal a 1.3 x 1.1 x 0.7 cm white rubbery mass with a SAVI scout clip and a heart biopsy clip. This mass was 0.6 cm from the anterior margin, 1.4 cm from the posterior margin, 3.5 cm from the lateral margin, 1.1 cm from the medial margin, and over 1.0 cm from the superior to inferior margins, and the remainder of the cut surface was yellow, lobulated with little to no fibrous tissue. Approximately 15 cm² of the total breast tissue was mobilized into the cavity to reduce the defect created by the operation and prevent nipple retraction. The final pathology resulted in AME.

## Discussion

Adenomyoepithelial tumors of the breast are very rare and account for less than 0.5% of all breast tumors [1]. It predominantly affects women over 50 and can be challenging to identify due to the clinical and diagnostically variable presentation [2]. Most AMEs are benign; however, malignant transformation can arise from either the epithelial or myoepithelial components [3]. It can present on clinical exam as a solitary palpable mass or on routine screening mammography. Most of the literature surrounding AMEs comes from case reports or small case series, which have postulated a potential link with breast cancer, either concomitant or remotely diagnosed [2,4,5]. However, the cause of this observed link is not fully understood, and further rigorous studies would be required to establish an association. 

The imaging appearance of AMEs is variable. In our patient, the mass was not palpable and was initially detected as a focal asymmetry on routine screening mammography. This mass was subsequently characterized sonographically as a round, hypoechoic mass with indistinct margins. On mammography, it most commonly presents as a round or lobulated mass with circumscribed or indistinct margins and a mean size of 2.0-2.5 cm [4]. Breast Imaging Reporting & Data System (BI-RADS) classification varied from 2 to 4 in the absence of secondary suspicious lesions [6]. On ultrasound, benign AMEs typically present as oval, circumscribed, hypoechoic masses; however, there are reports of irregular shape, indistinct margins, and complex cystic and solid echotexture. Peripheral and central vascularity on color Doppler is a common finding that has not been shown to help differentiate benign from malignant AMEs. Although MRI is not often required for the diagnosis, one study found most benign AMEs to present as round, isointense, homogenously enhancing nodules on T1-weighted imaging [7]. Contrast-enhanced mammography is a new tool that could be utilized in the future, but imaging features of AMEs in this modality have yet to be characterized. Imaging findings that could suggest malignant AMEs are not reliable or specific but could include poorly defined margins and posterior acoustic shadowing on sonography [8].

AMEs also present a diagnostic challenge for pathologists. Clues pointing toward a diagnosis of AME include large, spindle-shaped or epithelioid, clear cell myoepithelium surrounding epithelial-lined space [9]. When this lesion is suspected, immunohistochemistry is frequently utilized to confirm its components. Myoepithelial components are identified through the positivity of cytokeratin (CK) 5/6 antibodies, calponin, p63, smooth muscle actin, smooth muscle myosin, caldesmon, cd10, and S100 protein [10]. Morphologic features indicating malignant transformation include nuclear atypia, increased mitotic activity, and in particular atypical mitotic figures, necrosis, and an infiltrative growth pattern [11].

The differential diagnosis of AMEs includes fibroadenoma, intraductal papilloma, tubular adenoma, apocrine adenosis, sclerosing adenosis, and carcinoma. Misdiagnosis can be due to non-specific imaging findings and indistinct margins that make biopsy technically challenging.

The recommended treatment for AME is wide surgical excision to reduce local recurrence and exclude malignancy. If margins are positive, the simple excisional biopsy may need to be followed with complete excision, lumpectomy, or mastectomy. At present, lymph node biopsy is not routinely conducted for AME or AME with malignant transformation, irrespective of the presence or absence of metastasis evidence. Chemotherapy and radiotherapy use have only been reported for a few aggressive, recurrent tumors [4,12].

The risk of recurrence in AMEs has been a critical point of discussion in recent literature. In a study featuring a median follow-up of 55 months post-surgery, the five-year overall survival rate and disease-free survival rate were reported to be 87.5% and 91.7%, respectively [13]. Among the participants, two out of 15 cases experienced a recurrence, showcasing local recurrence and lung metastasis as potential complications. These numbers align with another recent study utilizing the National Cancer Database, which included 110 women diagnosed with AME. This larger study presented an expected five-year overall survival rate of 74.4% [14]. Unfortunately, due to limitations in the database, this study was unable to evaluate other critical outcomes, such as disease-free survival and locoregional recurrence rates. While the existing data provide some insight, a more comprehensive understanding of recurrence risks requires more detailed investigation, including the evaluation of disease-free survival rates and local recurrence.

## Conclusions

AME is a rare primary breast tumor that can be benign or malignant and has varied morphological features. On mammography, it most commonly presents as a round or lobulated mass with circumscribed or indistinct margins. Although certain non-specific imaging features have been reported, a final diagnosis requires a representative biopsy sample and histopathological analysis. The differential diagnosis of AME includes fibroadenoma, intraductal papilloma, tubular adenoma, apocrine adenosis, sclerosing adenosis, and carcinoma. Our case highlights the difficulty in diagnosing this lesion for both radiologists and pathologists alike. It also underlines the importance of wide local excision with negative margins in the presence of diagnostic uncertainty, which corresponds with the current recommended treatment for AME to prevent recurrence.
